# The influence of the sacrificial agent nature on transformations of the Zn(OH)_2_/Cd_0.3_Zn_0.7_S photocatalyst during hydrogen production under visible light†

**DOI:** 10.1039/c9ra08833d

**Published:** 2020-01-08

**Authors:** Dina V. Markovskaya, Svetlana V. Cherepanova, Evgeny Yu. Gerasimov, Angelina V. Zhurenok, Aleksandra V. Selivanova, Dmitry S. Selishchev, Ekaterina A. Kozlova

**Affiliations:** Boreskov Institute of Catalysis SB RAS Lavrentieva Ave., 5 Novosibirsk 630090 Russia kozlova@catalysis.ru +7-383-333-16-17 +7-383-333-16-17; Novosibirsk State University Pirogova Str., 2 Novosibirsk 630090 Russia

## Abstract

Photocatalysts based on zinc hydroxide and a solid solution of CdS and ZnS were prepared *via* the precipitation method and used for photocatalytic hydrogen production from aqueous solutions of inorganic (Na_2_S/Na_2_SO_3_) and organic (ethanol) sacrificial agents. The photocatalysts were tested in cyclic experiments for hydrogen evolution and studied using X-ray diffraction (XRD), UV-Vis diffuse reflectance spectroscopy, high-resolution transmission electron microscopy (HRTEM), energy-dispersive X-ray spectroscopy (EDX), and X-ray photoelectron spectroscopy (XPS) techniques. Different transformations of the β-Zn(OH)_2_ co-catalyst were observed in the presence of inorganic and organic sacrificial agents; namely, ZnS was formed in Na_2_S/Na_2_SO_3_ solution, whereas the formation of ε-Zn(OH)_2_ was detected in solution with ethanol. The composite Zn(OH)_2_/Cd_1−*x*_Zn_*x*_S photocatalysts have great potential in various photocatalysis processes (*e.g.*, hydrogen production, CO_2_ reduction, and the oxidation of organic contaminants) under visible light.

## Abstract 2

The nature of the sacrificial agent affects the transformations of a Zn(OH)_2_ co-catalyst during photocatalytic hydrogen production.

## Graphical abstract

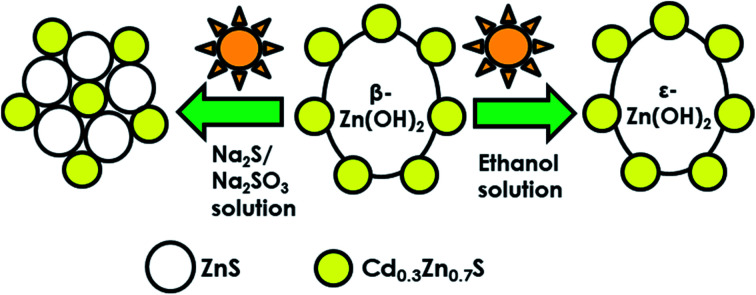

## 1. Introduction

Photocatalytic hydrogen evolution is known to be an environmentally friendly approach to the synthesis of H_2_ for various energy applications.^1,2^ From a practical point of view, the photocatalysts for hydrogen production should be active under visible light, because the solar radiation spectrum has *ca.* 40% of photons in the visible region of the spectrum.^3^ The materials based on CdS,^4^ ZnS,^4^ and their solid solution Cd_1−*x*_Zn_*x*_S (ref. 5) have great promise for this purpose. Unfortunately, the activity and stability of the Cd and Zn sulfide photocatalysts are quite low owing to the intense recombination of charge carriers and photocorrosion. The deposition of co-catalysts, such as oxides,^6–9^ hydroxides^10–12^ or other sulfides,^12–17^ can substantially improve the target parameters of the photocatalyst and enhance its activity. The photocatalysts based on the composition of Cd_1−*x*_Zn_*x*_S and Zn(OH)_2_ show a higher activity and stability compared to Cd_1−*x*_Zn_*x*_S alone and are attractive for the photocatalytic hydrogen evolution from water–alcohol solutions.^18^

In the 1950s, G. K. Boreskov stated that the chemical and phase composition of the heterogeneous catalyst was changed during the reaction owing to its interaction with the reagents.^19^ Studying the effect of the reagent nature on the catalyst composition and its catalytic activity is an important task from a fundamental point of view to discover the mechanism of the catalytic action. In a previous study,^18^ we demonstrated that the activation of the Zn(OH)_2_/Cd_0.3_Zn_0.7_S photocatalyst occurs during the photocatalytic hydrogen production from both the Na_2_S/Na_2_SO_3_ and ethanol aqueous solutions. This study aims to investigate in detail the changes in the composition of the Zn(OH)_2_/Cd_0.3_Zn_0.7_S photocatalyst during its activation in this process and to establish the role of the sacrificial agent on the transformation of the photocatalyst. In this paper, we show that for the Na_2_S/Na_2_SO_3_ aqueous solution the enhancement in the catalytic activity of the Zn(OH)_2_/Cd_0.3_Zn_0.7_S composite is due to the sulfurization of Zn(OH)_2_, while in the aqueous solution of ethanol the activation of the photocatalyst is associated with the formation of ε-Zn(OH)_2_. These results indicate great potential for Zn(OH)_2_/Cd_1−*x*_Zn_*x*_S photocatalysts, because a rather simple technique can be used for their synthesis, and for different sacrificial agents the co-catalyst would transform to the desired form *in situ* during the process of hydrogen evolution and would provide a high photocatalytic activity.

## 2. Experimental

### 2.1. Photocatalyst synthesis

The *y*% Zn(OH)_2_/Cd_0.3_Zn_0.7_S photocatalysts with a Zn(OH)_2_ weight content (*y*) from 10 to 30% were prepared *via* the precipitation of Zn(OH)_2_ by sodium hydroxide from an aqueous solution of zinc nitrate Zn(NO_3_)_2_, which contains suspended Cd_0.3_Zn_0.7_S nanoparticles, and was performed in accordance with the technique described in our previously published paper.^18^ The *y*% ZnS/Cd_0.3_Zn_0.7_S photocatalysts with a ZnS weight content (*y*) from 0.5 to 10% were prepared *via* the precipitation of ZnS by sodium sulfide from an aqueous solution of zinc nitrate Zn(NO_3_)_2_, which contains suspended Cd_0.3_Zn_0.7_S nanoparticles. Also, β-Zn(OH)_2_ was also prepared by a simple interaction of Zn(NO_3_)_2_ with NaOH followed by soft drying for several days. Note that the hydrogen evolution from the ethanol solution was a slow process, and to increase the photocatalytic activity in these experiments, the composite photocatalysts were additionally modified with platinum (1 wt%). Pt deposition was performed *via* the chemical reduction of H_2_PtCl_6_ with NaBH_4_ according to the previously published technique.^18^ The Pt-loaded photocatalysts are named in this paper as 1% Pt/*y*% Zn(OH)_2_/Cd_0.3_Zn_0.7_S. All of the reagents used during the synthesis were of analytic grade and were used as supplied without further purification.

### 2.2. Photocatalyst characterization

The photocatalysts were characterized before and after the catalytic tests using X-ray diffraction (XRD), UV-Vis diffuse reflectance spectroscopy (UV-Vis DRS), and transmission electron microscopy (TEM) techniques. The XRD patterns were recorded on a D8 Advance diffractometer (Bruker, Germany) using Cu Kα radiation. The scanning was performed in the 2*θ* range from 15 to 65° with a step of 0.05° and an acquisition time of 10 s at each point. The peak deconvolution was carried out using two Gaussian functions, and the composition of Cd_1−*x*_Zn_*x*_S was determined from the angular positions of the functions. The content of the ε-Zn(OH)_2_ phases was calculated with the TOPAS package (General Profile and Structure Analysis Software for Powder Diffraction Data, Bruker AXS GmBH, Germany). The weight content of Cd_0.3_Zn_0.7_S was assumed to be a constant. The hydrozincite (Zn_5_(CO_3_)_2_(OH)_6_) and β-Zn(OH)_2_ phases were not suitable for quantitative analysis with the TOPAS package owing to their imperfect structure and insufficient data. Therefore, their total content was estimated to be the difference between 100% and the calculated contents of Cd_1−*x*_Zn_*x*_S and ε-Zn(OH)_2._ The UV-Vis diffuse reflectance spectra of the photocatalysts were recorded at room temperature in the range of 250–850 nm with a resolution of 1 nm using a Cary 300 UV-Vis spectrophotometer from Agilent (USA) equipped with a DRA-30I diffuse reflectance accessory. Special pre-packed polytetrafluoroethylene (PTFE) from Agilent (USA) was used as a reflectance standard. The high resolution TEM (HRTEM) images were obtained with a JEM-2010 transmission electron microscope (JEOL, Japan) operated at an accelerating voltage of 200 kV which provides a lattice resolution of 0.14 nm. The local elemental composition was analyzed using a Phoenix energy-dispersive spectrometer (EDAX, USA) equipped with a Si(Li) detector with an energy resolution of 130 eV. X-ray photoelectron spectroscopy (XPS) measurements were performed using a SPECS photoelectron spectrometer (SPECS Surface Nano Analysis GmbH, Germany) equipped with an XR-50M X-ray source, a FOCUS-500 ellipsoidal crystal monochromator, and a PHOIBOS-150 hemispherical electron energy analyzer. The valence band and core-level spectra were obtained using monochromatic Al Kα radiation.

### 2.3. Photocatalytic activity measurement

The synthesized catalysts were studied for photocatalytic hydrogen evolution from an aqueous solution of inorganic or organic sacrificial agents according to the technique described elsewhere.^18^ In the case of the inorganic agent, 50 mg of *y*% Zn(OH)_2_/Cd_0.3_Zn_0.7_S photocatalyst was suspended in 100 mL of 0.1 M Na_2_S/0.1 M Na_2_SO_3_ aqueous solution, purged with argon for 30 min, and illuminated with a 450-LED (30 W, China). In the case of the organic donor, 50 mg of 1% Pt/*y*% Zn(OH)_2_/Cd_0.3_Zn_0.7_S was suspended in the solution, which contained 10 mL of ethanol, 90 mL of water, and 400 mg of NaOH. Then, the suspension was purged with argon for 30 min and illuminated with a 450-LED (30 W, China). The amount of evolved hydrogen was measured with a Khromos GCh-1000 gas chromatograph (Khromos, Russia) equipped with a zeolite column and a thermal conductivity detector. Argon was used as the carrier gas. The photocatalytic activity of the pristine Cd_0.3_Zn_0.7_S and synthesized composites was estimated as the rate of hydrogen evolution (μmol min^−1^). Note that the photocatalytic activity of the samples without any sacrificial agents was very low (*e.g.* 0.008 ± 0.002 μmol min^−1^ for Cd_0.3_Zn_0.7_S).

## 3. Results and discussion

Co-catalysts in composite photocatalysts may provide a substantial increase in the rate of photocatalytic hydrogen evolution from aqueous solutions of various sacrificial agents. The photocatalysts based on the composition of Cd_1−*x*_Zn_*x*_S and Zn(OH)_2_ show a high activity in this process. Earlier, we studied the photocatalytic activity of the *y*% Zn(OH)_2_/Cd_0.3_Zn_0.7_S and 1% Pt/*y*% Zn(OH)_2_/Cd_0.3_Zn_0.7_S composites for hydrogen production from aqueous solutions of Na_2_S/Na_2_SO_3_ and ethanol, respectively.^18^ The reaction rate increased as the Zn(OH)_2_ content was increased up to 10–20 wt% owing to the formation of heterojunctions between the components of the photocatalyst. In the case of the Na_2_S/Na_2_SO_3_ sacrificial agent, the maximum reaction rate was observed for the 20% Zn(OH)_2_/Cd_0.3_Zn_0.7_S sample. In the case of the ethanol sacrificial agent, a much lower rate of hydrogen production was observed, and the composites were additionally modified with platinum (1 wt%) to increase the photocatalytic activity. For ethanol as a sacrificial agent, the maximum reaction rate was observed for the 1% Pt/10% Zn(OH)_2_/Cd_0.3_Zn_0.7_S sample. In addition to the fact that Zn(OH)_2_ itself is not active in hydrogen production under visible light (Table 1), it may be considered as an active component of the composite photocatalyst.^18^ The level of the conduction band (CB) for Zn(OH)_2_ is close to −0.3 V *versus* normal hydrogen electrode (NHE), whereas for Cd_0.3_Zn_0.7_S, which is a light absorbing phase, the CB level is located at −0.6 V *versus* NHE. Therefore, the photogenerated electrons can transfer from the sulfide to the hydroxide nanoparticles,^2^ and the increased activity of the Zn(OH)_2_/Cd_0.3_Zn_0.7_S composites is due to the heterojunctions between the components of the photocatalyst and the improved separation of the charge carriers.

**Table 1:** Activities of photocatalysts over several runs of hydrogen evolution

Sample	Reaction rate (μmol min^−1^) achieved during the corresponding run			
	1	2	3	4
**0.1 M Na**_**2**_ **S/0.1 M Na**_**2**_ **SO**_**3**_ **solution**				
Cd_0.3_Zn_0.7_S^a^	3.2 ± 0.3	3.3 ± 0.3	2.8 ± 0.3	2.4 ± 0.2
10% Zn(OH)_2_/Cd_0.3_Zn_0.7_S^a^	3.3 ± 0.2	4.3 ± 0.4	5.1 ± 0.4	5.6 ± 0.3
20% Zn(OH)_2_/Cd_0.3_Zn_0.7_S^a^	3.5 ± 0.4	5.5 ± 0.5	7.4 ± 0.7	6.8 ± 0.7
30% Zn(OH)_2_/Cd_0.3_Zn_0.7_S^a^	3.1 ± 0.3	5.3 ± 0.5	6.2 ± 0.6	5.7 ± 0.6
0.5% ZnS/Cd_0.3_Zn_0.7_S^a^	3.4 ± 0.2	Experiments not carried out		
1% ZnS/Cd_0.3_Zn_0.7_S^a^	3.7 ± 0.3	4.2 ± 0.4	4.4 ± 0.5	4.0 ± 0.4
5% ZnS/Cd_0.3_Zn_0.7_S^a^	2.9 ± 0.3	Experiments not carried out		
10% ZnS/Cd_0.3_Zn_0.7_S^a^	1.9 ± 0.2			
ZnS^a^	0.30 ± 0.03			
Zn(OH)_2_^a^	No hydrogen is detected	0.14 ± 0.01	0.11 ± 0.03	0.14 ± 0.02

**10 vol% ethanol aqueous solution**				
1% Pt/Zn(OH)_2_^b^	No hydrogen is detected			
1% Pt/Cd_0.3_Zn_0.7_S^b^	0.48 ± 0.05	0.36 ± 0.04	0.24 ± 0.02	0.12 ± 0.01
1% Pt/10% Zn(OH)_2_/Cd_0.3_Zn_0.7_S^b^	0.94 ± 0.09	1.4 ± 0.1	2.3 ± 0.2	2.7 ± 0.3
1% Pt/30% Zn(OH)_2_/Cd_0.3_Zn_0.7_S^b^	0.10 ± 0.01	0.12 ± 0.01	0.63 ± 0.06	0.71 ± 0.07

a Hydrogen was produced from 0.1 M Na_2_S/0.1 M Na_2_SO_3_ solution.

b Hydrogen was produced from 10 vol% ethanol aqueous solution.

At the same time for both agents, a further increase in the content of Zn(OH)_2_ to greater than 10–20 wt% led to a decrease in the hydrogen production rate. The band gaps of Cd_0.3_Zn_0.7_S and Zn(OH)_2_ are ∼2.7 and 5.1 eV, respectively, and only Cd_0.3_Zn_0.7_S can be activated under visible light. Therefore, the low activity of the composite at a high content of Zn(OH)_2_ is due to a decrease in the amount of the Cd_0.3_Zn_0.7_S phase, which can absorb visible light.

Based on the points mentioned above, the composite Zn(OH)_2_/Cd_0.3_Zn_0.7_S photocatalysts with a content of Zn(OH)_2_ from 10 to 30% were selected for the experiments and detailed investigations in this study. The *y*% Zn(OH)_2_/Cd_0.3_Zn_0.7_S and 1% Pt/*y*% Zn(OH)_2_/Cd_0.3_Zn_0.7_S composites were tested in the cyclic experiments for hydrogen production with two sacrificial agents (Na_2_S/Na_2_SO_3_ and ethanol) under the same conditions to compare the transformation mechanism. For both sacrificial agents, the pristine Cd_0.3_Zn_0.7_S and 1% Pt/Cd_0.3_Zn_0.7_S samples lost their catalytic activity after several runs of hydrogen production (Table 1). This behavior may be due to the self-oxidation of the sulfide surface by the photogenerated holes.^20^ In contrast, the strong activation of the composite Zn(OH)_2_/Cd_0.3_Zn_0.7_S photocatalysts was achieved during three catalytic runs for both the inorganic and organic sacrificial agents (Table 1).

We have previously shown that the activation of 1% Pt/*y*% Zn(OH)_2_/Cd_0.3_Zn_0.7_S photocatalysts in the case of hydrogen evolution from aqueous ethanol solution is caused by the transformation of β-Zn(OH)_2_ to ε-Zn(OH)_2_.^18,21^ The linear correlation between the reaction rate and the content of ε-Zn(OH)_2_ in the composite photocatalyst was found.^22^ Based on these results, the same transformation may be suggested for the photocatalytic hydrogen evolution from the Na_2_S/Na_2_SO_3_ aqueous solution. To check this suggestion, a detailed analysis of the photocatalyst composition during long-term hydrogen evolution was performed using several characterization methods. It should be noted that the 20% Zn(OH)_2_/Cd_0.3_Zn_0.7_S and 1% Pt/10% Zn(OH)_2_/Cd_0.3_Zn_0.7_S samples had the maximum activity for Na_2_S/Na_2_SO_3_ and ethanol sacrificial agents, respectively, but these samples were not suitable for correct identification using XRD analysis owing to the low content of Zn(OH)_2_. This was the reason for the selection of the 30% Zn(OH)_2_/Cd_0.3_Zn_0.7_S and 1% Pt/30% Zn(OH)_2_/Cd_0.3_Zn_0.7_S samples with a Zn(OH)_2_ content of 30 wt% for detailed analysis of the phase transformation. To support the findings and to extend them to the samples with a reduced content of Zn(OH)_2_, the 10% Zn(OH)_2_/Cd_0.3_Zn_0.7_S and 20% Zn(OH)_2_/Cd_0.3_Zn_0.7_S samples were also tested in four consecutive runs for hydrogen evolution from the Na_2_S/Na_2_SO_3_ aqueous solution. However, only the phase compositions of the fresh samples and the samples after the fourth photocatalytic run were analyzed.

**Fig. 1:**
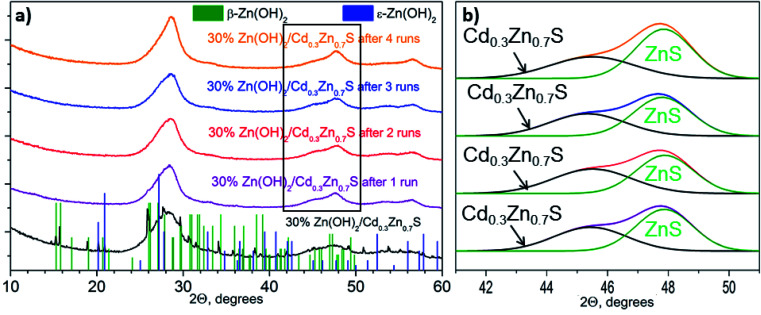
(a) XRD patterns of 30% Zn(OH)_2_/Cd_0.3_Zn_0.7_S before and after photocatalytic hydrogen production from aqueous 0.1 M Na_2_S/0.1 M Na_2_SO_3_ solutions; and (b) deconvolutions of the second peak from the obtained patterns.

Fig. 1a shows the XRD patterns for the 30% Zn(OH)_2_/Cd_0.3_Zn_0.7_S photocatalyst before and after the hydrogen evolution runs. Three broad peaks located at 25–30, 42–50, and 52–60° are observed for the fresh photocatalyst. These peaks can be attributed to the Cd_1−*x*_Zn_*x*_S solid solution of *x* ∼ 0.7. Narrow peaks for the β-Zn(OH)_2_ and ε-Zn(OH)_2_ phases are also observed. At the same time, no peaks attributed to Zn(OH)_2_ can be identified in the XRD patterns for the 30% Zn(OH)_2_/Cd_0.3_Zn_0.7_S photocatalysts after several runs of hydrogen evolution (Fig. 1a). An amorphization of zinc hydroxide or a decrease in its content during the runs may be the reason for this. Additionally, after the first run, the broad peaks attributed to the Cd_1−*x*_Zn_*x*_S solid solution became asymmetrical owing to the appearance of a signal from the ZnS phase (Fig. 1b). The formation of ZnS may result from the sulfurization of Zn(OH)_2_ in the Na_2_S/Na_2_SO_3_ aqueous solution, because the area of the ZnS peak grew after each run of hydrogen evolution (Fig. 1b). The same behavior was observed for the 10% Zn(OH)_2_/Cd_0.3_Zn_0.7_S and 20% Zn(OH)_2_/Cd_0.3_Zn_0.7_S photocatalysts after the fourth run (Fig. S1 in the ESI†). This statement also supported the experiments on Zn(OH)_2_ alone, after four runs in the Na_2_S/Na_2_SO_3_ aqueous solution the pristine Zn(OH)_2_ underwent partial sulfurization (see Fig. S2 in ESI†). Unfortunately, we cannot exactly calculate the ratio of Cd_0.3_Zn_0.7_S to ZnS, because both phases have a disordered structure.

**Fig. 2:**
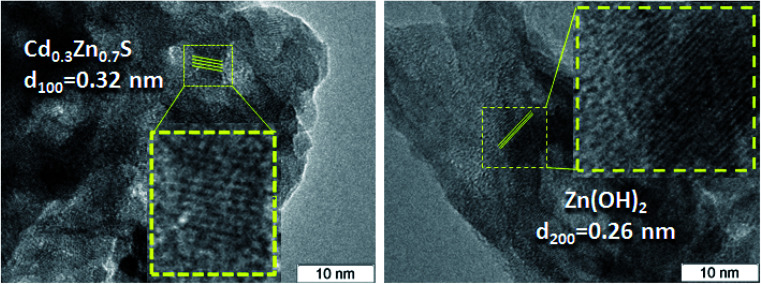
HRTEM images of a fresh 30% Zn(OH)_2_/Cd_0.3_Zn_0.7_S sample.

**Fig. 3:**
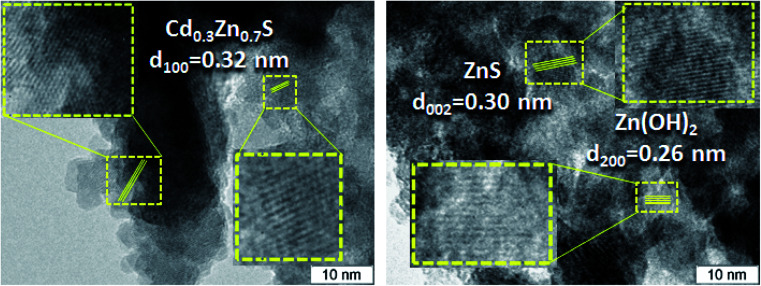
HRTEM images of a 30% Zn(OH)_2_/Cd_0.3_Zn_0.7_S sample after two photocatalytic runs.

The TEM and HRTEM images of the 30% Zn(OH)_2_/Cd_0.3_Zn_0.7_S before and after two irradiation runs were used to evaluate the degree of transformation of Zn(OH)_2_ to ZnS (Fig. 2 and 3). Fig. 2 shows that the fresh 30% Zn(OH)_2_/Cd_0.3_Zn_0.7_S photocatalyst consists of two different phases, namely, the Cd_0.3_Zn_0.7_S solid solution (*d*_100_ = 0.32 nm) and Zn(OH)_2_ (*d*_200_ = 0.32 nm). Fig. 3 shows that the same phases are presented in the photocatalyst after two runs of hydrogen evolution. In addition to these phases, the lattice fringe of 0.30 nm can be attributed to the (002) plane of ZnS. It indicates that zinc hydroxide is present in the sample, but the size of the Zn(OH)_2_ particle is much smaller.

**Fig. 4:**
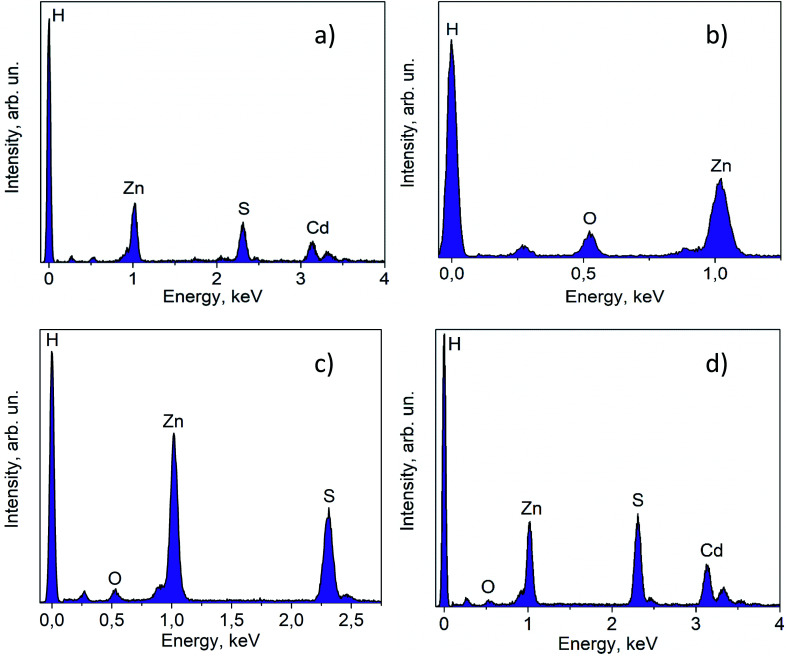
EDX spectra for fresh 30% Zn(OH)_2_/Cd_0.3_Zn_0.7_S (a) and (b); and for 30% Zn(OH)_2_/Cd_0.3_Zn_0.7_S after two irradiation runs (c) and (d).

The lattice fringes corresponding to Cd_1−*x*_Zn_*x*_S and ZnS are close to each other, and to confirm the presence of the ZnS phase the samples were additionally investigated using the EDX technique (Fig. 4). The lines of Cd, Zn, and S are observed in the EDX spectrum of the fresh 30% Zn(OH)_2_/Cd_0.3_Zn_0.7_S sample, while the line for O is very low (Fig. 4a). These data are attributed to the solid solution of Cd_1−*x*_Zn_*x*_S. Fig. 4b shows the EDX spectrum for the domains depicted in Fig. 2 (right) and confirms the presence of zinc hydroxide only. The lines, which can be attributed to Zn and S (Fig. 4c), as well as to Zn, Cd, and S (Fig. 4d), were observed for the sample after two runs of hydrogen evolution. In both cases, the content of oxygen was very low. Therefore, the area in Fig. 4c can be attributed to the ZnS phase, while the area in Fig. 4d corresponds to Cd_1−*x*_Zn_*x*_S. The data from the EDX analysis for the studied samples are summarized in Table 2. They confirm the presence of two types of domains, as mentioned above. It should be noted that the O content for the fresh photocatalyst varied from 9 to 54 at%, while for the sample after two runs of hydrogen evolution this value was in the range of 3–33 at%. Simultaneously, the S content increased during the long-term photocatalytic hydrogen production. The lowest S content was equal to 2 at% for the fresh photocatalyst and 25 at% for the photocatalyst after long-term hydrogen production. The highest S content also grew from 42 to 52 at%. This increase in the S content with a simultaneous decrease in the O content confirms the partial sulfurization of zinc hydroxide during the photocatalytic hydrogen production.

**Table 2:** EDX-analysis of several TEM images of the composite photocatalysts

Number of the analyzed area	Atomic content of corresponding element, %			
	Cd	Zn	S	O
**30% Zn(OH)**_**2**_ **/Cd**_**0.3**_ **Zn**_**0.7**_ **S**				
1	4	45	32	19
2	6	37	28	29
3	3	40	36	21
4	21	30	40	9
5	2	45	3	50
6	1	43	2	54
7	13	32	42	13

**30% Zn(OH)**_**2**_ **/Cd**_**0.3**_ **Zn**_**0.7**_ **S after two irradiation runs**				
8	1	42	37	20
9	1	40	40	19
10	2	36	52	10
11	1	41	25	33
12	20	25	51	4
13	1	40	49	10
14	22	22	52	4

**Fig. 5:**
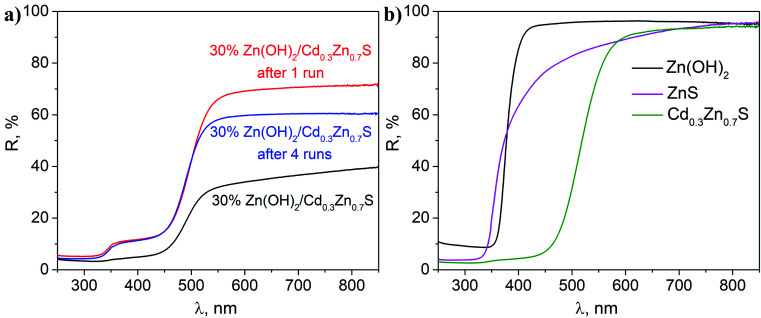
DRS analysis of (a) a fresh 30% Zn(OH)_2_/Cd_0.3_Zn_0.7_S sample and 30% Zn(OH)_2_/Cd_0.3_Zn_0.7_S samples after the first and fourth irradiation runs; and (b) single-phase Cd_0.3_Zn_0.7_S, Zn(OH)_2_, and ZnS.

Fig. 5a shows the diffuse reflectance spectra of the fresh photocatalyst and the sample after the first and fourth runs of hydrogen evolution. The fresh photocatalyst had a spectrum that is typical of materials based on a Cd_0.3_Zn_0.7_S solid solution.^4^ No peaks that are typical of Zn(OH)_2_ were observed for this photocatalyst. The spectra of the composite samples after the first and fourth runs were similar, which corresponds to the light absorption by the Cd_0.3_Zn_0.7_S phase (Fig. 4). According to the spectra for individual phases (Fig. 5b), an additional shoulder at ∼340 nm in these spectra can be attributed to the ZnS phase.^23^ The DRS data confirm the sulfurization of the co-catalyst and completely agrees with the data from other methods.

Therefore, the sulfurization of the Zn(OH)_2_/Cd_0.3_Zn_0.7_S photocatalysts occurs during the photocatalytic hydrogen production from the Na_2_S/Na_2_SO_3_ aqueous solution under visible light. The XRD patterns of the 30% Zn(OH)_2_/Cd_0.3_Zn_0.7_S sample after the second to the fourth photocatalytic runs in Fig. 1b are quite similar and confirm the presence of ZnS and Cd_0.3_Zn_0.7_S only. Also, Table 1 shows that the rate of hydrogen evolution almost doubles after the first run, and then it does not change significantly. Based on these results, we can conclude that the transformation of β-Zn(OH)_2_ to ZnS is quite a fast process. In addition to the fact that zinc hydroxide nanoparticles are present in the sample (Fig. 3), their amount is probably small. ZnS is known to have a weak absorption in the visible region,^23^ and both Cd_0.3_Zn_0.7_S and ZnS can be activated under visible light. However, we believe that the heterojunctions between ZnS and Cd_0.3_Zn_0.7_S are probably realized in the multiphase sample and are responsible for the improvement of the photocatalytic activity. The kinetic data (Table 1) confirms this statement, because the hydrogen production rate for the 20 or 30% Zn(OH)_2_/Cd_0.3_Zn_0.7_S samples, which transforms to the form of ZnS/Cd_0.3_Zn_0.7_S during the reaction, is higher than the rate for Cd_0.3_Zn_0.7_S (3.2 μmol min^−1^) or ZnS (0.3 μmol min^−1^).

To confirm the high activity of the ZnS/Cd_1−*x*_Zn_*x*_S heterostructures, the photocatalytic activity of the *y*% ZnS/Cd_0.3_Zn_0.7_S samples, prepared *via* a simple deposition of ZnS onto the surface of Cd_0.3_Zn_0.7_S, was also studied and is listed in Table 1. The reaction rate slightly increased as the ZnS content was increased up to 1 wt%. The further increase in the ZnS content led to a decrease in the reaction rate (Table 1). This observation confirms that the formation of ZnS from Zn(OH)_2_*in situ* during the process of hydrogen production is beneficial to a high photocatalytic activity compared to the direct synthesis of the ZnS/Cd_0.3_Zn_0.7_S composites. This is probably due to the closer contact between the formed nanoparticles in the first case.

**Fig. 6:**
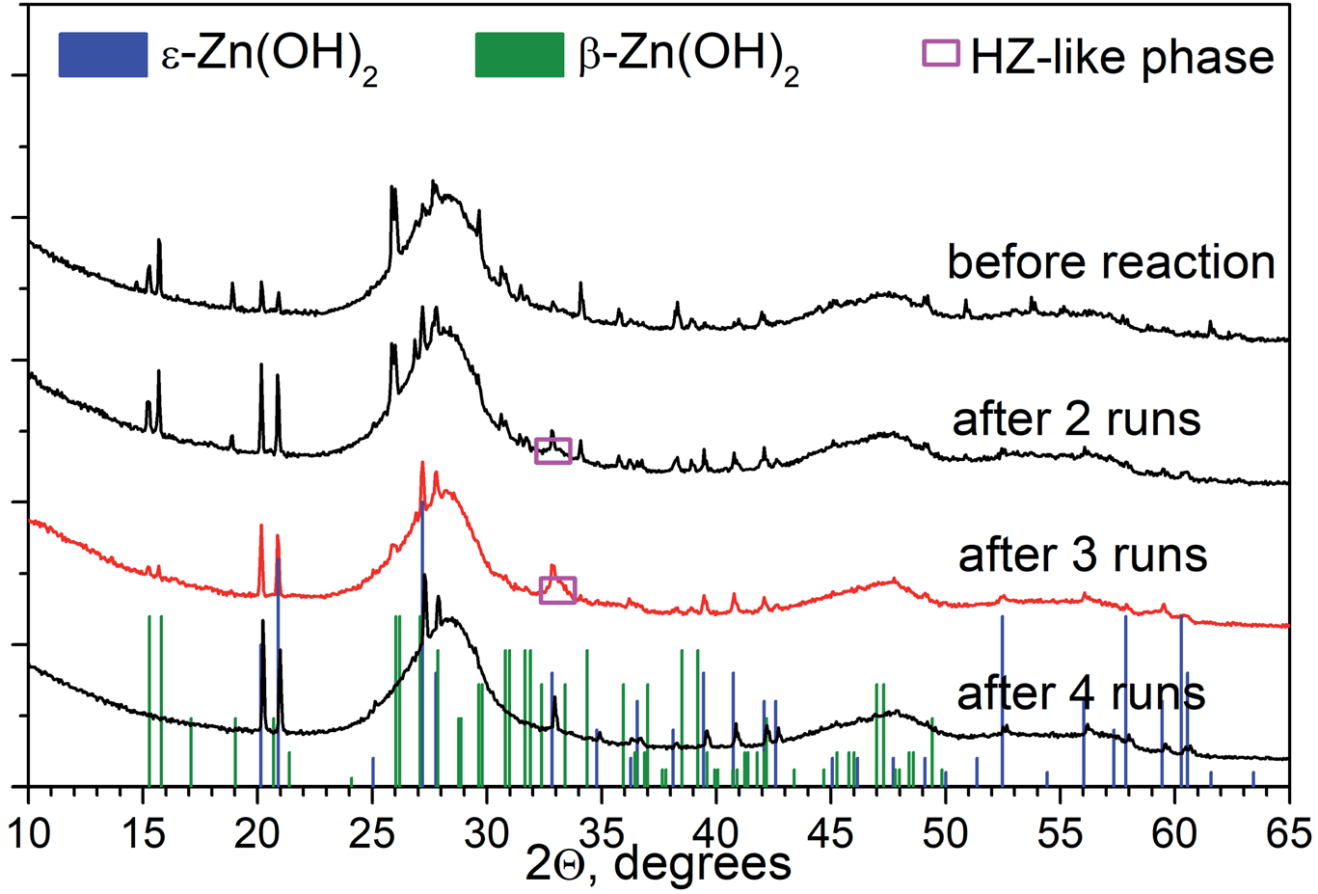
XRD patterns of 1% Pt/30% Zn(OH)_2_/Cd_0.3_Zn_0.7_S before and after photocatalytic hydrogen production from aqueous ethanol solutions.

**Fig. 7:**
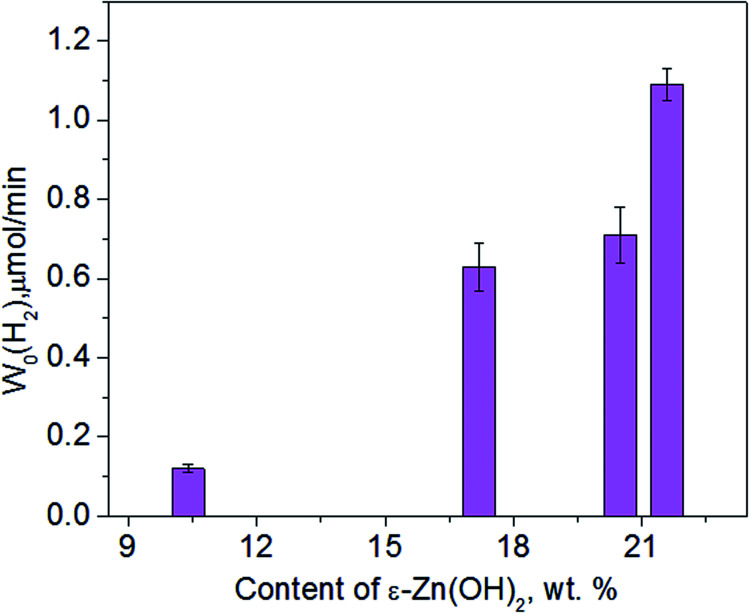
Dependence of the photocatalytic hydrogen production rate on the ε-Zn(OH)_2_ weight percentage obtained in ethanol solution in the presence of 1% Pt/30% Zn(OH)_2_/Cd_0.3_Zn_0.7_S.

In addition to Na_2_S/Na_2_SO_3_, we also checked the data for ethanol as a sacrificial agent, and the transformations of the 1% Pt/30% Zn(OH)_2_/Cd_0.3_Zn_0.7_S photocatalyst under irradiation in an aqueous solution of ethanol were studied in detail. Fig. 6 shows the XRD patterns of 1% Pt/30% Zn(OH)_2_/Cd_0.3_Zn_0.7_S before and after the second to the fourth runs of hydrogen evolution. Cd_0.3_Zn_0.7_S and a mixture of the β-Zn(OH)_2_ and ε-Zn(OH)_2_ phases were detected in the composition of the fresh photocatalyst. During the photocatalytic hydrogen production, the intensity of the peaks, which correspond to β-Zn(OH)_2_, decreased, whereas the intensity of the ε-Zn(OH)_2_ peaks increased in contrast, and the reaction rate also increased (see Fig. 7 and Table 1). Quantitative analysis of the XRD data confirms this observation (see Table 3). 11 wt% β-Zn(OH)_2_ and 11 wt% ε-Zn(OH)_2_ was detected in the fresh photocatalyst, whereas only the ε-Zn(OH)_2_ phase (22 wt%) was detected in the photocatalyst after four runs of hydrogen evolution. Additionally, the hydrozincite-like phase Zn_5_(CO_3_)_2−*x*_(OH)_6+2*x*_·*y*H_2_O, which is referred to as HZ, was detected after the first run, but it completely disappeared at the end of the fourth run. The HZ phase may be formed during the wet stage of the synthesis owing to the presence of dissolved CO_2_ in solution and/or during drying of the samples in air.^24^ We have previously shown that the presence of the ε-Zn(OH)_2_ phase is beneficial for H_2_ production, whereas both β-Zn(OH)_2_ and HZ are inactive in this process.^22^ As further support for this statement, the reaction rate of the hydrogen production in this study monotonically increased as the content of ε-Zn(OH)_2_ increased (see Fig. 7).

**Table 3:** Composition of the 1% Pt/30% Zn(OH)_2_/Cd_0.3_Zn_0.7_S photocatalyst estimated using the XRD technique during photocatalytic hydrogen evolution

Run number	Cd_0.3_Zn_0.7_S, wt%	ε-Zn(OH)_2_, wt%	β-Zn(OH)_2_ + hydrozincite, wt%
0^a^	78	11	11
2	78	18	4
3	78	21	1
4	78	22	0

a No peaks from the hydrozincite-like phase were observed for the fresh photocatalyst.

The most active photocatalyst, 1% Pt/30% Zn(OH)_2_/Cd_0.3_Zn_0.7_S, was also characterized using an XPS technique before and after four runs of hydrogen evolution (Table 4). After the long-term photocatalytic process, the surface ratio of cadmium to zinc fell from 3.9 to 1.8. At the same time, the surface ratio of sulfur to oxygen increased in contrast. Therefore, β-Zn(OH)_2_, which according to the synthesis technique probably covers the surface of Cd_0.3_Zn_0.7_S, transforms to ε-Zn(OH)_2_ with a high crystalline size and releases from the surface of the Cd_0.3_Zn_0.7_S sulfide. The surface ratio and oxidation state of the deposited platinum (Pt^0^/Pt^2+^) were similar before and after four runs (Table 4). This result indicates that the change in activity of the composite photocatalysts is caused by bulk transformations rather than the surface transformations.

**Table 4:** Surface composition of the studied photocatalysts and Pt 4f_7/2_ binding energies, calculated using the XPS technique

Sample	[Zn]/[Cd]	[O]/[Cd + Zn]	[S]/[Cd + Zn]	[Pt]/[Zn + Cd]	Pt 4f_7/2_	
					Pt^0^	Pt^2+^
1% Pt/30% Zn(OH)_2_/Cd_0.3_Zn_0.7_S (fresh)	3.9	1.91	0.48	0.014	71.1 (75%)	72.4 (25%)
1% Pt/30% Zn(OH)_2_/Cd_0.3_Zn_0.7_S (after four runs)	1.8	1.15	0.69	0.017	71.1 (80%)	72.4 (20%)

Therefore, the transformation of 1% Pt/β-Zn(OH)_2_/Cd_0.3_Zn_0.7_S to 1% Pt/ε-Zn(OH)_2_/Cd_0.3_Zn_0.7_S leads to a significant growth in the H_2_ production rate from the ethanol aqueous solution. It is important to note that up to a seven-fold increase in the rate of the photocatalytic hydrogen evolution was achieved in the case of the ethanol donor during the four catalytic runs, whereas for the hydrogen evolution from the Na_2_S/Na_2_SO_3_ aqueous solution the rate increased only twice (Table 1). Therefore, the heterojunctions between ε-Zn(OH)_2_ and Cd_0.3_Zn_0.7_S can be concluded to be more efficient than those between ZnS and Cd_0.3_Zn_0.7_S.

To summarize, the zinc hydroxide co-catalyst in the Zn(OH)_2_/Cd_0.3_Zn_0.7_S composite photocatalysts undergoes sulfurization during the photocatalytic hydrogen production from Na_2_S/Na_2_SO_3_ solution, whereas during the hydrogen evolution from ethanol aqueous solution the transformation of the β-Zn(OH)_2_ co-catalyst to ε-Zn(OH)_2_ was realized. A significant increase in the activity accompanies both types of transformations. The highest hydrogen production rates are 3200 μmol g^−1^ h^−1^ for 1% Pt/10% Zn(OH)_2_/Cd_0.3_Zn_0.7_S photocatalyst (ethanol) and 8900 μmol g^−1^ h^−1^ for the 20% Zn(OH)_2_/Cd_0.3_Zn_0.7_S photocatalyst (Na_2_S/Na_2_SO_3_) which are comparable with recently published data (see Table 5).^25–35^ Therefore, the sacrificial agent as a reaction media plays a key role in the photocatalyst transformation and affects its catalytic activity (see graphical abstract). This is a very promising result, because it is possible to synthesize a composite photocatalyst by a rather simple technique, and the co-catalyst could be tuned *in situ* during the process of photocatalytic hydrogen evolution. In our previous works, we made a significant efforts to obtain a system with zinc hydroxide for the desired ε-modification.^21^ The present study shows that the initial state of the photocatalyst is not as important for its activity.

**Table 5:** Comparison of the photocatalytic activities over sulfide-based catalysts

Photocatalyst	Sacrificial agent	Light source	Catalytic activity, μmol g^−1^ h^−1^	Ref.
**Inorganic sacrificial agents**				
ZnS/hydrogel	0.1 M Na_2_S, 0.1 M Na_2_SO_3_	Xe lamp	400	1
30% CdS/Zn_2_GeO_4_	0.35 M Na_2_S, 0.25 M Na_2_SO_3_	Xe lamp, *λ* > 420 nm	1720	2
0.3% NiS/30% CdS/TiO2	0.35 M Na_2_S, 0.25 M Na_2_SO_3_	Xe lamp, *λ* > 420 nm	2159	3
3D CdS/graphene	0.5 M Na_2_S, 0.5 M Na_2_SO_3_	Xe lamp, *λ* > 400 nm	2310	4
3% Pt/10% CdS/Ti^3+^/TiO_2_	0.35 M Na_2_S, 0.25 M Na_2_SO_3_	Xe lamp, *λ* > 420 nm	4474	5
5% NC@Mo_2_N/CdS	0.25 M Na_2_S, 0.35 M Na_2_SO_3_	Xe lamp, *λ* > 400 nm	7294	6
**20% Zn(OH)**_**2**_ **/Cd**_**0.3**_ **Zn**_**0.7**_ **S**	**0.1 M Na**_**2**_ **S, 0.1 M Na**_**2**_ **SO**_**3**_	**450-LED**	**8900**	**This paper**
0.5% Pt/CdS/Cu_2_ZnSnS_4_	0.4 M Na_2_S, 0.3 M Na_2_SO_3_	Xe lamp, *λ* > 420 nm	11 540	7

**Organic sacrificial reagents**				
0.2% NiS/CdS	Lignin, lactic acid	Xe lamp, *λ* > 400 nm	1086	8
CdS/MoS_2_ QDs/ZnIn_2_S_4_	20% vol. lactic acid	Xe lamp, *λ* > 420 nm	2108	9
5% MoS_2_/Co_0.2_Cd_0.8_S	10% vol. lactic acid	Xe lamp, *λ* > 400 nm	2836	10
**1% Pt/10% Zn(OH)**_**2**_ **/Cd**_**0.3**_ **Zn**_**0.7**_ **S**	**10% vol. ethanol**	**450-LED**	**3200**	**This paper**
Co_4_S_3_/CdS	10% vol. lactic acid	Xe lamp, *λ* > 420 nm	5893	11

## 4. Conclusions

In this study, we have shown for the first time that the nature of the sacrificial agent strongly affects the transformation of the composite photocatalyst during photocatalytic hydrogen production. The composites, which consist of a Cd_0.3_Zn_0.7_S photocatalyst and a Zn(OH)_2_ co-catalyst, are active during the photocatalytic hydrogen evolution under visible light with both inorganic (Na_2_S/Na_2_SO_3_) and organic (ethanol) sacrificial agents, but in Na_2_S/Na_2_SO_3_ solution, the Zn(OH)_2_ co-catalyst transforms to zinc sulfide, whereas in ethanol solution, β-Zn(OH)_2_ transforms to ε-Zn(OH)_2_. Both transformations are accompanied by a significant increase in the photocatalytic activity that is probably due to heterojunctions in the formed pairs of ZnS/Cd_0.3_Zn_0.7_S and ε-Zn(OH)_2_/Cd_0.3_Zn_0.7_S, respectively.

## Conflicts of interest

There are no conflicts of interest to declare.

## Supplementary Material

RA-010-C9RA08833D-s001
