# Supplementary material for: The influence of the sacrificial agent nature on transformations of the Zn(OH)2/Cd0.3Zn0.7S photocatalyst during hydrogen production under visible light
Source: RSC Adv. 2020 Jan 8;10(3):1341–50. doi: 10.1039/c9ra08833d (PMC9048281; doi:10.1039/c9ra08833d)
Supplement: RA-010-C9RA08833D-s001 [file RA-010-C9RA08833D-s001.pdf]

Electronic Supplementary Material (ESI) for RSC Advances.
This journal is © The Royal Society of Chemistry 2019

SUPPORTING INFORMATION

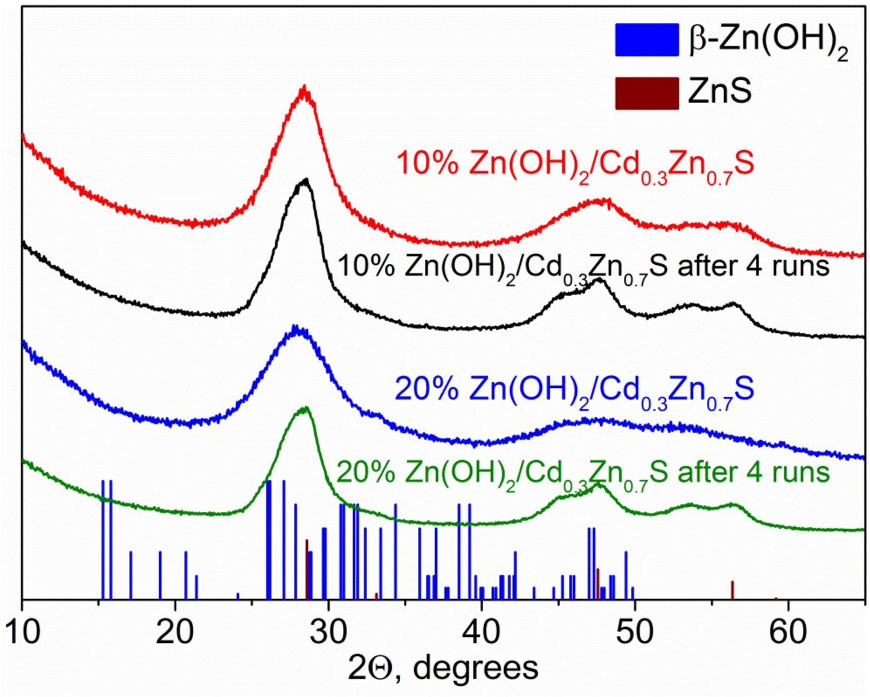

Fig. S1. XRD patterns of 10% $Zn(OH)_2/Cd_{0.3}Zn_{0.7}S$ and 20% $Zn(OH)_2/Cd_{0.3}Zn_{0.7}S$ before and after photocatalytic hydrogen production from aqueous 0.1 M Na_2S/0.1 M Na_2SO_3 solution

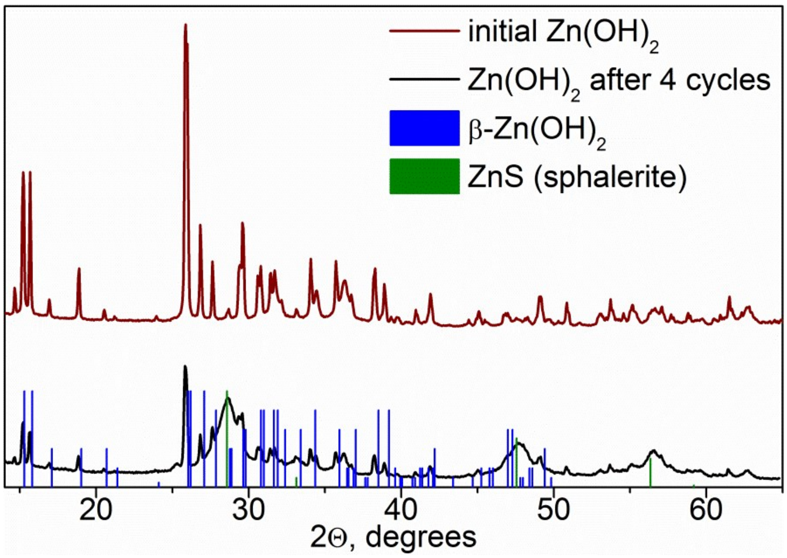

Fig. S2. XRD patterns of blank $Zn(OH)_2$ before and after photocatalytic hydrogen production from aqueous 0.1 M Na_2S/0.1 M Na_2SO_3 solution
